# *Streptococcus pneumoniae* non-susceptibility and outpatient antimicrobial prescribing rates at the Alaska Native Medical Center

**DOI:** 10.3402/ijch.v72i0.22297

**Published:** 2013-12-17

**Authors:** Ryan W. Stevens, Jay Wenger, Lisa Bulkow, Michael G. Bruce

**Affiliations:** 1 Alaska Native Medical Center, Anchorage, AK, USA; 2 Arctic Investigations Program, Division of Preparedness and Emerging Infections, National Center for Emerging and Zoonotic Infectious Disease, Centers for Disease Control and Prevention (CDC), Anchorage, AK, USA

**Keywords:** *Streptococcus pneumoniae*, invasive pneumococcal disease, antibiotic resistance, antibiotic prescribing, susceptibility, Alaska Natives, Alaska

## Abstract

**Background:**

American Indian/Alaska Native (AI/AN) people suffer substantially higher rates of invasive pneumococcal disease (IPD) than the general US population. We evaluated antimicrobial prescribing data and their association with non-susceptibility in *Streptococcus pneumoniae* causing IPD in AI/AN people between 1992 and 2009.

**Methods:**

Antimicrobial use data were gathered from the electronic patient management system and included all prescriptions dispensed to Alaska Native patients aged 5 years and older from outpatient pharmacies at the Alaska Native Medical Center (ANMC). Antimicrobial susceptibility data were gathered from pneumococcal isolates causing IPD among Anchorage Service Unit AI/AN residents aged 5 years and older. Data were restricted to serotypes not contained in the pneumococcal vaccine (PCV7).

**Results:**

Over the study period, overall antimicrobial prescribing increased 59% (285/1,000 persons/year in 1992 to 454/1,000 persons per year in 2009, p<0.001). Trimethoprim/sulfamethoxazole prescribing increased (43/1,000 persons/year in 1992 to 108/1,000 persons/year in 2009, p<0.001) and non-susceptibility to trimethoprim/sulfamethoxazole in AI/AN patients ≥5 years of age increased in non-PCV7 serotypes (0–12%, p<0.05). Similarly, prescribing rates increased for macrolide antibiotics (46/1,000 persons/year in 1992 to 84/1,000 persons/year in 2009, p<0.05). We observed no statistically significant change over time in erythromycin non-susceptibility among non-PCV7 serotypes in AI/AN patients aged 5 years or greater (0–7%, p=0.087).

**Conclusion:**

Antimicrobial prescribing patterns of some antibiotics in the AI/AN population corresponded to increased antimicrobial resistance in clinical isolates. This study highlights the on-going threat of antimicrobial resistance, the critical importance of judicious prescribing of antibiotics and the potential utility of prescribing data for addressing this issue.

Streptococcus pneumoniae is associated with a high degree of morbidity and mortality in many countries worldwide and may account for as many as 1 million paediatric deaths annually in developing countries (1,2). This organism is one of the most common pathogens associated with respiratory tract infections (RTIs) and meningitis (1,3). American Indian and Alaska Native (AI/AN) people have had some of the highest reported rates of invasive pneumococcal disease (IPD) in the world (4). Increasing antimicrobial resistance in this community-acquired pathogen has been an area of concern over the previous 2 decades (1,4–6). Early initiation of appropriate antibiotic therapy for IPD has been linked to improved outcomes (7). Empiric antimicrobial therapy for IPD is typically chosen before sensitivities of the infecting organism are identified; thus, clinicians and healthcare practitioners need to know the *S. pneumoniae* antimicrobial resistance patterns for their region and the state in general (6). While data from Europe exist on the impact of changes in prescribing rates on non-susceptibility patterns of *S. pneumoniae* causing IPD, data from the same population over time have seldom been evaluated within the United States (3,8–15). Evaluation of such data is especially critical in Alaska, where use of the 7-valent pneumococcal vaccine (PCV7), resulted in significant replacement with non-vaccine serotypes such as serotype 19A, which is often multidrug resistant (MDR) (16).

The objectives of this study were to characterize outpatient antimicrobial prescribing patterns for Alaska Native persons ≥5 years of age who received care at the Alaska Native Medical Center (ANMC) in Anchorage, Alaska and to evaluate the association between antimicrobial prescribing patterns and *S. pneumoniae* antimicrobial sensitivities in AI/AN in the Anchorage Service Unit.

## Methods

PCV7 was introduced to the routine paediatric vaccination schedule in Alaska in 2001, and subsequently PCV7 serotype isolates became increasingly uncommon. Therefore, we restricted our analysis to non-PCV7 serotypes causing IPD in the Anchorage Service Unit from 1992 to 2009. Furthermore, due to the rarity of non-PCV7 serotypes among children <5 years of age, we restricted the analysis to children ≥5 years of age.

The Anchorage Service Unit covers a large area in Southeastern Alaska of approximately 107,413 miles^2^. The majority of Anchorage Service Unit residents live in the Municipality of Anchorage and the Matanuska Valley. The other urban areas within the Service Unit are Homer, Kenai, Kodiak, Soldotna, Seward, Unalaska and Valdez (http://www.ihs.gov/alaska/documents/hf/asu.pdf).

Data on antimicrobial usage were gathered from pharmacy prescription records using an electronic data system known as the Resource Patient Management System (RPMS) at ANMC in Anchorage, a 150-bed hospital and primary care facility that delivers care to AI/AN patients residing within the Anchorage Service Unit. ANMC is the only Indian Health Service (IHS) hospital in Anchorage, and, in conjunction with the associated outpatient clinics, provides care to most AI/AN patients residing in the Anchorage Service Unit. All prescriptions written for an oral antimicrobial agent (suspension, tablet or capsule) from a select group of antibiotic classes (trimethoprim/sulfamethoxazole, tetracyclines, macrolides, penicillins and cephalosporins) and dispensed from 1 of 4 outpatient pharmacies at ANMC to patients aged 5 years and older were included. We evaluated antimicrobial prescribing trends for Alaska Native patients residing in the Anchorage Service Unit (as defined by the IHS) who had been seen at an ANMC clinic. Patients were determined to be residing within the Anchorage Service Unit if their current address listed in the hospital prescription database was in any city within the Anchorage Service Unit. Prescription records were obtained from 1992 to 2009. Prescriptions were separated by antimicrobial class and totalled by year. The Alaska Area Institutional Review Board (IRB) approved this project.

We reviewed antimicrobial susceptibility data on IPD cases in AI/AN people ≥5 years of age living in the Anchorage Service Unit. Additionally, to compare *S. pneumoniae* non-susceptibility in this population to those found in non-Native Alaskans residing in the same geographical area, cases of IPD in non-AI/AN people living in the Anchorage Service Unit were reviewed. These data were obtained from the laboratory-based surveillance conducted by the Centers for Disease Control and Prevention, Arctic Investigations Program (16).

A case of IPD was defined as the presence of *S. pneumoniae* in a normally sterile site in a patient with signs and symptoms consistent with IPD. IPD isolates were tested for susceptibility to penicillin, erythromycin, tetracycline, ceftriaxone, cefotaxime and trimethoprim/sulfamethoxazole. Fluoroquinoles, as a class, were not included in the review since data on fluoroquinolone non-susceptibility were not available throughout the entire study period. Host race was classified as AI/AN or non-AI/AN. Due to the small number of total IPD cases reported in any given year, non-susceptibility data on *S. pneumoniae* isolates causing IPD were analyzed in 4- or 5-year time periods and were reported as a mean.

Organisms were classified as non-susceptible if they exhibited either full or intermediate resistance to the antimicrobial agent in question. Minimum inhibitory concentrations (MIC) used were in accordance with guidelines set by the Clinical and Laboratory Standards Institute (17). Although changes in these standards occurred between 1992 and 2009, the susceptibility levels shown in Table I were applied to all isolates in the study to provide a consistent measure of changes in MICs over time.

**Table I:** Minimum inhibitory concentration (MIC) endpoints used in this study (17)

Antimicrobial agent	Susceptible	Intermediate	Resistant
Penicillin	≤2	4	≥8
Cefotaxime/ceftriaxone	≤1	2	≥4
Tetracycline	≤2	4	≥8
Erythromycin	≤0.25	0.5	≥1
Trimethoprim/sulfamethoxazole	≤0.5	1–2	≥4

## Statistical analysis

Statistical tests performed in this study were completed using STATA^®^: Data Analysis and Statistical Software. For antimicrobial prescribing rates, the number of prescriptions dispensed from outpatient pharmacies at ANMC to Anchorage Service Unit AI/AN patients aged 5 years and older was totalled for each antimicrobial for each year of the study. Prescribing rates were calculated and presented as the number of prescriptions per 1,000 persons in the population per year. Weighted least squares and logit models were used to test for linear trends in antimicrobial prescribing patterns from 1992 to 2009. For all tests, statistical significance was defined as a p-value <0.05. Antimicrobial non-susceptibility rates were calculated by determining the percentage of isolates which were non-susceptible to the respective antimicrobial agents relative to the total number of available isolates for the given patient population. These rates are presented over the following time periods: 1992–1996, 1997–2000, 2001–2004 and 2005–2009. Trends were established using the Chi-square test for trends to compare rates of non-susceptibility over the different time periods of the study.

## Results

### Antimicrobial use data

From 1992 to 2009, a total of 292,717 (mean per year=16,262) oral antimicrobial prescriptions for either penicillins, cephalosporins, macrolides, tetracyclines or trimethoprim/sulfamethoxazole were dispensed from the outpatient pharmacies at ANMC to patients aged 5 years or older residing within the Anchorage Service Unit. Of the total number of dispensed prescriptions, penicillins accounted for 40%; trimethoprim/sulfamethoxazole 21%; cephalosporins 17%; macrolides 14% and tetracyclines 8%. Between 1992 and 2009, the population of AI/AN 5 years of age or older living in the Anchorage Service Unit grew from 35,129 to 42,647.

**Fig. 1:**
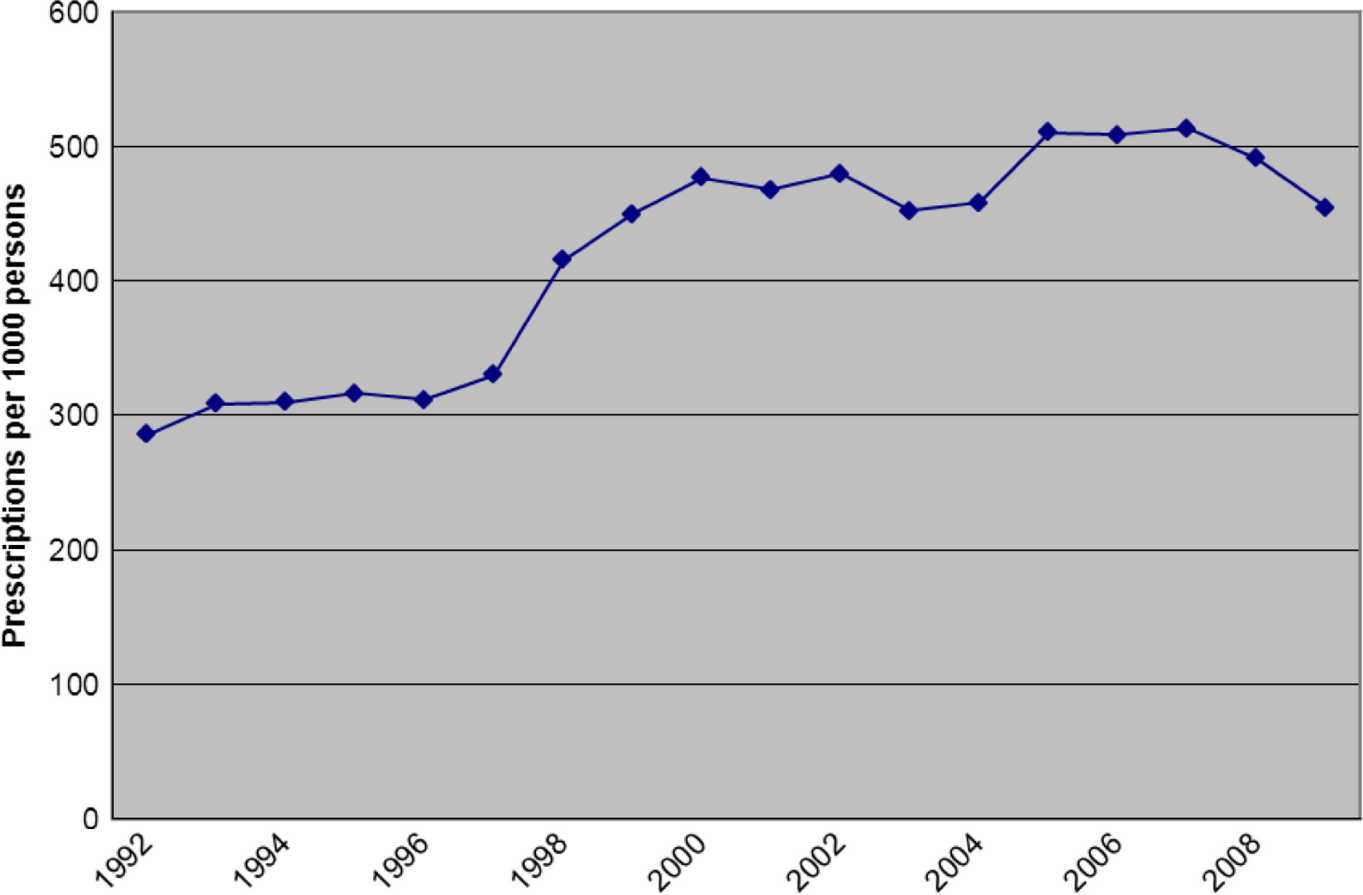
Total antibiotic usage (Ceph+PCN+TET+macrolides+TMP/SMX) American Indian/Alaska Native persons ≥5 years of age (1992–2009).

Overall, antimicrobial prescribing increased by 59% from 285 prescriptions per 1,000 persons per year to 454 prescriptions per 1,000 persons per year between 1992 and 2009 (p<0.001). Figure 1 illustrates changes in antimicrobial prescribing for all antimicrobials over the time period studied.

### Invasive pneumococcal disease

Between 1992 and 2009, a total of 247 cases of IPD in Anchorage Service Unit AI/AN persons ≥5 years of age were reported and 641 cases were reported for non-AI/AN persons of these ages residing in the Anchorage Service Unit. Of the total 247 AI/AN IPD cases, 206 (85%) were caused by non-PCV7 serotypes. In the non-AI/AN group, a total of 423 (66%) out of the 655 cases of IPD were caused by non-PCV7 serotypes. The later years of the study produced the largest number of cases of IPD caused by non-PCV7 serotypes as compared to other time intervals with 48.5% (n=100) and 49.4% (n=209) of non-PCV7 cases being reported between 2005 and 2009 for AI/AN and non-AI/AN people, respectively. We were unable to look at differences in prescribing patterns between AI/AN and non-AI/AN patients due to the lack of prescribing data for the non-AI/AN population; however, we were able to compare antimicrobial susceptibility patterns over time between AI/AN and non-AI/AN persons which did not differ significantly.

### Prescribing and antimicrobial non-susceptibility

#### Trimethoprim/sulfamethoxazole

**Fig. 2:**
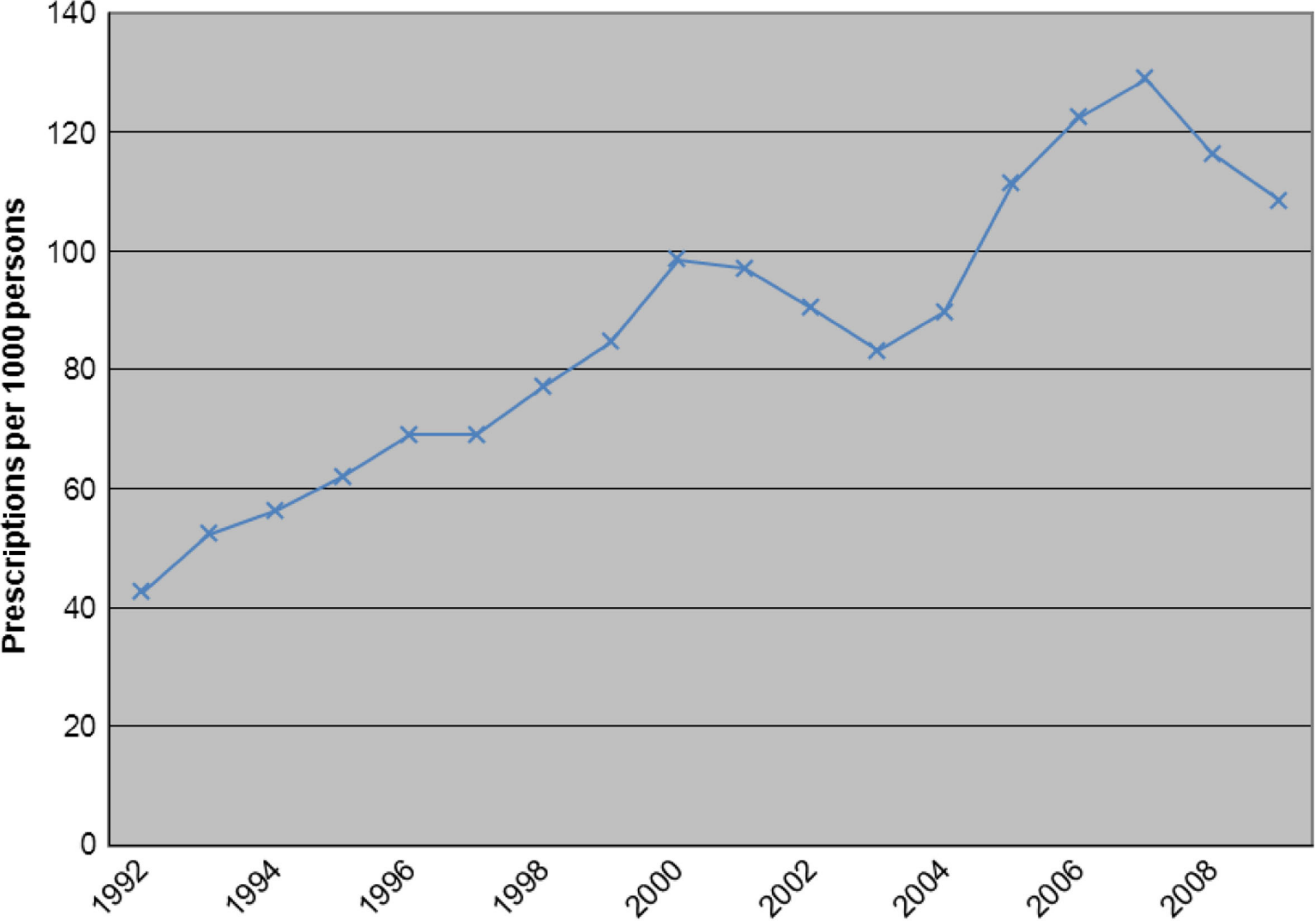
Trimethoprim/sulfamethoxazole prescription rate per 1,000 American Indian/Alaska Native persons ≥5 years of age (1992–2009).

The antimicrobial prescribing rate for trimethoprim/sulfamethoxazole increased 151% from 43 prescriptions per 1,000 persons in 1992 to 108 per 1,000 persons in 2009 (p<0.001, Fig. 2).

**Fig. 3:**
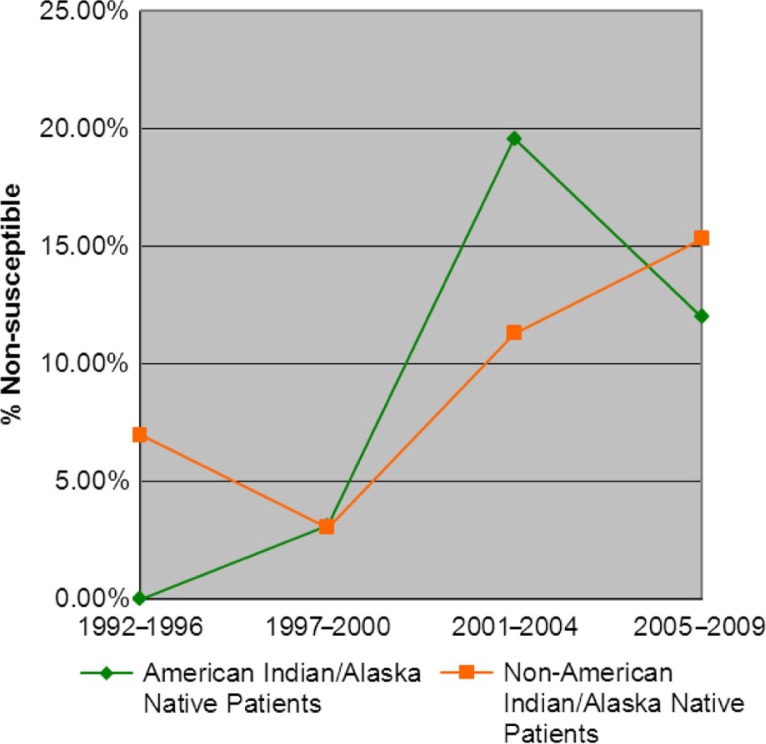
TMP/SMX non-susceptibility in IPD isolates (non-PCV7 serotypes) from patients ≥5 years of age (1992–2009).

In AI/AN patients, over the entire study period, 22 isolates were non-susceptible (14 fully resistant and 8 intermediately resistant) and the rate of non-susceptibility increased from 0 to 12% (p=0.044, Fig. 3). The first trimethoprim/sulfamethoxazole non-susceptible IPD isolate during the study period was isolated from an AI/AN person in 2000. This isolate was only intermediately resistant; however, the first fully resistant isolate was collected 1 year later in 2001. Among isolates from non-AI/AN persons, the non-susceptibility to trimethoprim/sulfamethoxazole increased from 7 to 15% from 1992–1996 to 2005–2009, respectively (p=0.014, Fig. 3); over the entire period, 47 isolates were non-susceptible (25 fully resistant and 22 intermediately resistant).

#### Macrolides

**Fig. 4:**
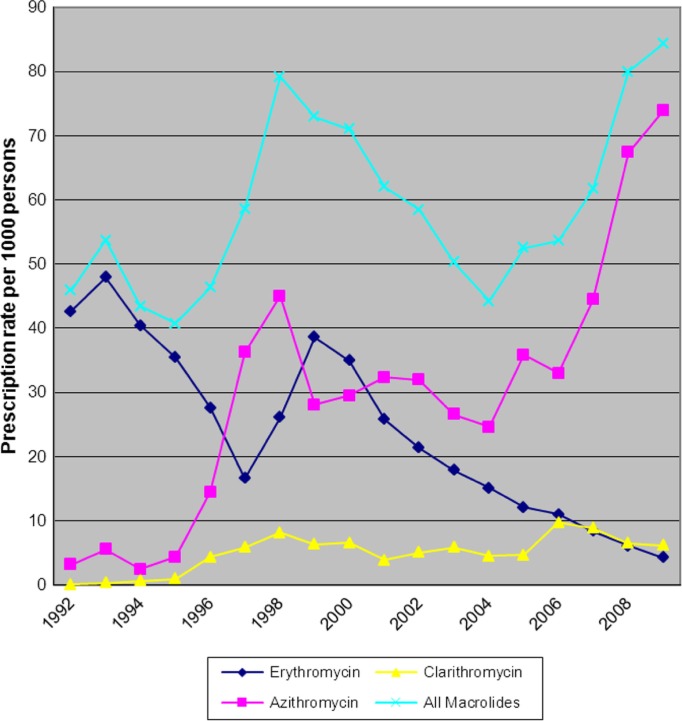
Macrolide prescription rate per 1,000 American Indian/Alaska Native persons ≥5 years of age (1992–2009).

Prescribing for macrolide antibiotics increased from 46 prescriptions per 1,000 persons in 1992 to 84 per 1,000 persons in 2009, an 83% increase (p=0.041, Fig. 4). The increase in macrolide prescribing was primarily due to a large increase in prescribing for azithromycin, increasing from 3 prescriptions per 1,000 persons in 1992 to 74 per 1,000 persons in 2009 (2,400% increase, p<0.001, Fig. 4). Erythromycin use decreased between 1992 and 2009 from 43 to 4 prescriptions per 1,000 persons, respectively (p<0.001). Although the prescribing rate of clarithromycin increased by 600% between 1992 and 2009, the actual number of prescriptions was minimal with rates increasing from 0 to 6 prescriptions per 1,000 persons (p=0.008).

**Fig. 5:**
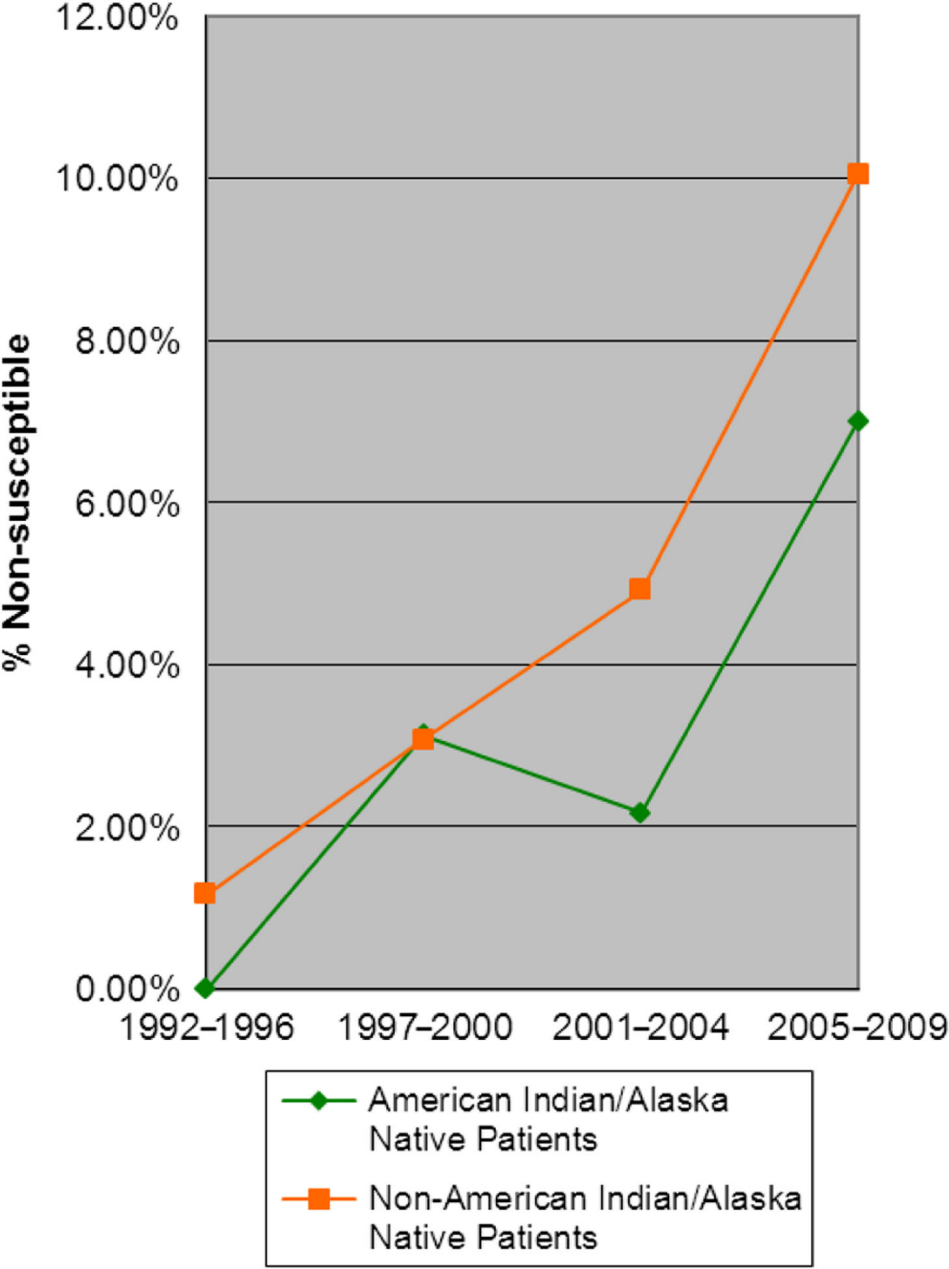
Erythromycin non-susceptibility in IPD isolates (non-PCV7 serotypes) from patients ≥5 years of age (1992–2009).

Changes in erythromycin non-susceptibility (0–7%) over the time period (1992–2009) among non-PCV7 serotypes in IPD isolates from AI/AN persons were not statistically significant (p=0.087, Fig. 5). Nine non-susceptible isolates were identified, all fully resistant to erythromycin. The highest numbers of non-susceptible isolates were isolated in the last year of the study (2009), when 4 of 24 isolates (17%) were fully resistant. Among IPD isolates from non-AI/AN, the non-susceptibility rate increased from 1 to 10% (p=0.002). Of the 27 non-susceptible isolates, 26 isolates were reported as fully resistant. The one intermediately resistant isolate was collected in 1997, and it was the only non-susceptible isolate gathered in non-AI/AN persons that year.

#### Tetracyclines

Tetracycline prescribing increased by 31% from 1992 to 2009, with annual rates increasing from 32 prescriptions per 1,000 persons to 42 per 1,000 persons (p<0.001). Tetracycline-class prescriptions included doxycycline (85%) and tetracycline hydrochloride (15%).

Three of 206 isolates collected from AI/AN persons showed non-susceptibility to tetracycline antibiotics (overall non-susceptibility of 1.5% between 1992 and 2009). The non-susceptible isolates were collected in 1997, 2005 and 2006, and all showed full resistance to tetracycline. Tetracycline non-susceptibility demonstrated no statistically significant changes (0% in 1992–1996 to 2% in 2005–2009, p=0.66). In non-AI/AN persons, 9 of 423 cases of IPD caused by non-PCV7 serotype were non-susceptible (7 showing full resistance and 2 intermediate resistance) for an overall non-susceptibility rate of 2%. The non-susceptibility rate demonstrated no statistically significant changes (0–3.4%) between 1992–1996 and 2005–2009, respectively (p=0.065).

#### Cephalosporins

Prescribing increased from 40 prescriptions per 1,000 persons in 1992 to 59 prescriptions per 1,000 persons in 2009, a 48% increase (p<0.001). Oral cephalosporins included cephalexin, cefdinir, cefixime, cefpodoxime and cefuroxime.

*Streptococcus pneumoniae* susceptibility data were available for both cefotaxime and ceftriaxone; however, not all isolates were tested for both ceftriaxone; therefore, only cefotaxime is reported here. Only 1 of 206 isolates collected from AI/AN persons showed non-susceptibility to cefotaxime (an overall rate of 0.5%). This isolate was collected in 2007 and was fully resistant to the antibiotic. In non-AI/AN cases, 8 isolates were non-susceptible (6 intermediate and 2 fully resistant), and 7 of 8 were collected between 2005 and 2009. The non-susceptibility rate among non-AI/AN cases demonstrated no statistically significant changes (1–3%) between 1992–1996 and 2005–2009, respectively (p=0.101).

#### Penicillins

In 1992, 124 penicillin prescriptions per 1,000 persons were dispensed and by 2009 this rate had increased by 30% to 161 prescriptions per 1,000 persons (p<0.001). The increase in prescribing was the sum of changes in the rate of prescribing of penicillin, amoxicillin, amoxicillin/clavulanate and other penicillin prescribing. Penicillins included in the other penicillin category included carbenicillin, dicloxacillin, ampicillin and cloxicillin. Between 1992 and 2009, the prescribing rate for amoxicillin/clavulanate rose from 6 prescriptions per 1,000 persons to 46 per 1,000 persons (670%, p<0.001). Prescribing for amoxicillin alone increased from 75 prescriptions per 1,000 persons in 1992 to 92 per 1,000 persons in 2009, a 22% increase (p<0.001). Penicillin prescribing alone demonstrated no statistically significant changes [29 prescriptions per 1,000 persons in 1992 to 21 per 1,000 persons in 2009 (−28%, p=0.28)]; however, the prescribing rate of other penicillins decreased significantly from 14 prescriptions per 1,000 persons in 1992 to 2 per 1,000 persons in 2009 (−86%, p<0.001).

The overall rate of penicillin non-susceptibility in isolates collected from AI/AN persons was 9.7%. Between 1992 and 2009, only one isolate showed full resistance to penicillin antibiotics and a total of 19 isolates showed intermediate resistance. Between 1992 and 1996, the penicillin non-susceptibility rate was 7%. By 2005–2009, the non-susceptibility rate was 11%, which was not statistically significantly different (p=0.408). In non-AI/AN persons, the overall rate of non-susceptibility to penicillin antibiotics was 10.4%. Non-susceptibility to penicillins increased to a statistically significant degree in non-AI/AN persons from 7% (1992–1996) to 14% (2005–2009, p=0.018).

## Discussion

Antimicrobial prescribing as a whole increased significantly during the study period. When further separated by antimicrobial class, statistically significant increases in prescribing were seen in every class of antibiotics evaluated for patients aged 5 years and older. The 2 classes of antibiotics which increased to the greatest degree were trimethoprim/sulfamethoxazole (151% increase) and the macrolides (83% increase). While data from the late 1990s and early 2000s suggested decreasing antimicrobial use in the general US population, these data show increasing antimicrobial use over the study period in an urban setting in Alaska and emphasize the importance of continued efforts to enhance judicious use of antimicrobials (3,18,19). During the same time period, non-susceptibility rates of IPD caused by non-PCV7 serotypes of *S. pneumoniae* increased for some of the antimicrobial agents evaluated. The only statistically significant increase in non-susceptibility among AI/AN IPD isolates was for trimethoprim/sulfamethoxazole (p=0.044). However, among non-AI/AN persons, statistically significant increases in trimethoprim/sulfamethoxazole (p=0.007), macrolides (p=0.002) and penicillin class (p=0.018) non-susceptibility were observed.

While the trend towards increased non-susceptibility for macrolides in AI/AN IPD isolates only approached statistical significance, (p=0.087), the patterns noted here suggest a correlation between the magnitude of increased use of specific antibiotics and the development of resistance to those specific antibiotics in community isolates of *Streptococcus pneumoniae* causing IPD. Non-susceptibility to antibiotics did not change significantly (penicillins, cephalosporins, tetracyclines) when the increase in rate of antimicrobial prescribing was relatively small (less than 50% over the study period), while non-susceptibility to macrolides (use increase of 83%) increased significantly in non-AI/AN with a strong trend in AI/AN persons, and non-susceptibility to trimethoprim/sulfamethoxazole (150% increase in use) increased significantly in both AI/AN and non-AI/AN populations.

It is important to note that our study looked at antimicrobial prescribing on a population level and not on an ambulatory clinic visit level. A previous study evaluated antimicrobial prescribing on both a population level and ambulatory visit level in AI/AN<18 years of age residing in the Anchorage area and found that visit-based antimicrobial prescribing rates remained stable between 1992 and 2004. This was accompanied by a significant increase in both the number of visits and antimicrobial prescribing on a population level, which took into account population growth over time (20). Therefore, although the visit-based antimicrobial prescribing level has remained stable, more visits have resulted in a higher volume of antimicrobial usage within the population. This principle may account for the large rise in antimicrobial prescribing in our study between 1997 and 2000 as a new and larger Alaska Native Tribal Health Consortium hospital was opened in May of 1997 in Anchorage. Use of the new hospital translated to higher volumes of clinic visits and therefore increased prescribing rates on a population level whereas visit-based prescribing rates may not have changed significantly. In this case, the end result is a higher volume of antimicrobials being dispensed into our study population over time yielding increased potential for selective pressure on resistant organisms.

Increased use is only one factor contributing to development of resistance. Duration of action may also play a role in the selection of resistant organisms. Long-acting macrolides, versus short-acting macrolides, may select for non-susceptible isolates of *S. pneumoniae* more easily through the possession of a longer selective window. Long-acting macrolides, such as azithromycin, maintain concentrations sufficient to slow or limit growth of susceptible organisms for longer periods of time; therefore, allowing for longer periods of selection for those organisms possessing traits that allow them to evade the antibacterial action of the agent (15). In our study, when the macrolide class of antibiotics was separated into specific agents, the increase in antimicrobial non-susceptibility appears to be largely due to the increase in azithromycin prescribing. It has been previously established that azithromycin, erythromycin and clarithromycin non-susceptibilities correlate closely, and as a result erythromycin non-susceptibility rates are an appropriate indicator of overall macrolide non-susceptibility (15). The Alexander Project, which included prescribing data from France, Germany, Italy, Spain, United Kingdom and the United States, found that increases in the prescribing rate of azithromycin were associated with increasing *S. pneumoniae* macrolide non-susceptibility rates (15). Prescribing trends found in this study were similar to those found in our study; increases in azithromycin and clarithromycin prescribing and decreases in erythromycin prescribing. They found that in the United States, overall macrolide prescribing rates decreased between 1986 and 1997; however, non-susceptibility rates increased between 1992 and 1996. This coincided with the use of long-acting macrolides such as azithromycin (3,15). In our study, azithromycin prescribing increased by 2,400% between 1992 and 2009 from 3 prescriptions per 1,000 persons to 74 prescriptions per 1,000 persons, respectively. Clarithromycin prescribing increased by 600% over the study period (p<0.001); however, it is unlikely that this agent's prescribing influenced the non-susceptibility trend to a large degree given that the actual prescribing rate was still very low (6/1,000 persons) in 2009.

The ANMC provided a unique practice setting for evaluating the temporal effects of prescribing on non-susceptibility within a population. In our study, antimicrobial prescribing rates were defined as the number of prescriptions dispensed from ANMC outpatient pharmacies to Anchorage Service Unit AI/AN patients (i.e. number of prescriptions per 1,000 persons per year). While this measure of antimicrobial prescribing does not account for patient non-compliance or antimicrobial acquisition by other means, and therefore may potentially be a suboptimal measure of the quantity of antimicrobial reaching the population, it provides more accurate estimates of the amount of antimicrobials reaching the population than estimates based on regional pharmaceutical purchasing data. Several studies have been published which examine the development of non-susceptibility as it pertains to outpatient antimicrobial prescribing; however, a majority of them define antimicrobial prescribing through the use of wholesale pharmaceutical purchasing data (11–14,21). Another strength of our study was the ability to correlate non-susceptibility patterns of Alaska Native persons with those of non-Native persons residing in the same geographic region. This allowed for confirmation that the non-susceptibility rates seen in Alaska Native persons are likely representative of the population of the geographical region as a whole.

Our study has several limitations. First, generalization of our data to other populations is not possible given that prescribing data were only available from outpatient pharmacies at ANMC and therefore the effect of antimicrobial prescribing on IPD non-susceptibility could only be evaluated in the AI/AN population. We did show that non-susceptibility rates of IPD isolates in non-AI/AN persons were similar to those of AI/AN people and suggested that similar trends in antimicrobial susceptibility may reflect similar antimicrobial prescribing practices in these groups; however, this would need to be verified with future research. Also, our study design did not fully capture antimicrobial use in the population since inpatient antimicrobial agents were not included nor was prescribing data for Anchorage Service Unit AI/AN patients who chose to seek care at facilities other than ANMC. Another limitation is the lack of the ability to account for multiple prescriptions for the same episode of an illness in one individual or multiple prescriptions for any given individual. This considered, it is possible that the distribution of antimicrobial prescriptions may be skewed by those patients who received several courses of antimicrobials in a given year or those prescribed multiple antibiotics for a single illness episode; thus, potentially making gross aggregate antimicrobial prescribing rates misleading. Additionally, antimicrobial rates for children <5 years of age were not accounted for in our study. Children are typically propagators of pneumococcal transmission and therefore antimicrobial usage in this patient population may potentially have the largest effect on pneumococcal resistance development in the population as a whole. It is important to note that, although we were able to identify a correlation between prescribing and non-susceptibility, particularly in trimethoprim/sulfamethoxazole, the design of the study does not allow for establishment of a causal relationship between increasing prescribing and increases in non-susceptibility.

The PCV7 vaccine was added to the standard paediatric vaccination schedule in Alaska in 2001. This vaccine covers serotypes 4, 6B, 9V, 14, 18C, 19F and 23F (22). The number of IPD cases caused by PCV7 serotypes was higher than non-PCV7 serotypes during the initial years of the study; however, following the introduction of the vaccine, non-PCV7 serotypes causing IPD increased in prevalence. Thus, by utilizing a group of serotypes that persisted throughout the entire study, we were able to examine the effect of antimicrobial pressure on non-susceptibility patterns.

## Conclusion

Antimicrobial prescribing rates in the AI/AN population are increasing for cephalosporin, penicillin, macrolide, tetracycline and sulfonamide antibiotics. Overall antimicrobial non-susceptibility of *S. pneumoniae* isolated from patients with IPD is also increasing. Non-susceptibility rates appear to have been similar for AI/AN and non-AI/AN patients over the 18-year study period. Large increases (>50%) in antimicrobial prescribing were correlated with increases in non-susceptibility whereas smaller increases in antimicrobial prescribing rates in AI/AN populations were associated with smaller, non-statistically significant increases in non-susceptibility. Our data are consistent with a role for azithromycin as the primary macrolide associated with the development of non-susceptibility. This study emphasizes the potential problems associated with antibiotic use and the importance of practitioner familiarity with antimicrobial prescribing patterns, local antibiotic resistance data and guidelines for judicious antibiotic use.
